# Whole Genome Sequencing Reveals Genetic Differences Between Symbiodiniaceae Populations Among Reproductively and Geographically Isolated *Acropora* Colonies in Western Australia

**DOI:** 10.1002/ece3.70771

**Published:** 2025-01-02

**Authors:** Sanna Y. Eriksson, Mikhail V. Matz, Peter D. Vize, Natalie L. Rosser

**Affiliations:** ^1^ School of Earth, Atmospheric and Life Sciences University of Wollongong Wollongong New South Wales Australia; ^2^ Climate Change Cluster (C3) University of Technology Sydney Broadway New South Wales Australia; ^3^ Department of Integrative Biology University of Texas at Austin Austin Texas USA; ^4^ Xenbase, Departments of Biological Sciences and Computer Science University of Calgary Calgary Alberta Canada; ^5^ Australian Institute of Marine Science Indian Ocean Marine Research Centre Crawley Western Australia Australia

**Keywords:** *Acropora cf. secale*, *Acropora millepora*, genetic variation, temporal reproductive isolation, whole genome sequencing

## Abstract

Significant genetic differentiation between Symbiodiniaceae populations in coral hosts can be induced by a range of factors including geography, latitude, depth, temperature and light utilisation. The conventional method of measuring Symbiodiniaceae diversity involving the ITS2 region of rDNA has several limitations, stemming from insufficient genetic resolution and the multi‐copy nature of the marker. This could be improved by using higher throughput whole genome sequencing to identify fine‐scale population genetic differences and provide new insight into factors influencing coral‐Symbiodiniaceae associations. The aim of this study was to investigate the genetic diversity of Symbiodiniaceae populations using low‐coverage whole genome sequencing in sympatric populations of *Acropora cf. secale* and allopatric populations of *Acropora millepora* that reproduce in different seasons in Western Australia. Genetic diversity of Symbiodiniaceae populations in these two species was examined using principal coordinates analysis and permutational analysis of variance. This analysis revealed that while all colonies were dominated by *Cladocopium*, there was a significant genetic difference between Symbiodiniaceae populations in both species. In *A. millepora* , this variation could be due to the latitudinal variation between populations or differences in reproductive seasonality, but in sympatric populations of *A. cf. secale*, genetic differences between Symbiodiniaceae populations were clearly aligned with the reproductive seasonality of the coral host. The use of whole genome sequencing improved the sensitivity to detect Symbiodiniaceae genetic population structure between coral populations, which increases our ability to identify genetic and potentially functional differences associated with variation in Symbiodiniaceae populations.

## Graphical abstract

In this study, we used a whole genome sequencing approach to determine whether reproductively and geographically isolated Acropora colonies in Western Australia harbour genetically different Symbiodiniaceae populations. We found that both allopatric and sympatric populations have significantly genetically different symbionts, potentially due to environmental or genetic factors.

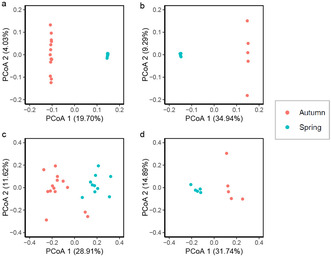

## 1 Introduction

Symbiodiniaceae is a family of unicellular photosynthetic microalgae that commonly form endosymbiotic relationships with reef‐building corals (LaJeunesse et al. 2018). Most broadcast‐spawning coral species obtain Symbiodiniaceae from their environment through horizontal transmission (Yakovleva et al. 2009) and rely on the photosynthesis of these acquired Symbiodiniaceae as the source of the majority of their energy (Muscatine 1990; Rädecker et al. 2018). Notably, there is a high level of genetic diversity in the Symbiodiniaceae family, which is accompanied by extensive physiological and ecological variation between different Symbiodiniaceae species. Thus, coral‐Symbiodiniaceae associations can be strongly influenced by both host identity (LaJeunesse et al. 2010; Lewis, Chan, and LaJeunesse 2019) and environmental factors (Cooper et al. 2011; Chen et al. 2019; Matias et al. 2022).

Among geographically separated coral populations, there is often significant genetic differentiation between Symbiodiniaceae populations due to limited dispersal and microevolutionary forces such as genetic drift and mutation (van Oppen et al. 2001; Thornhill et al. 2017; Ziegler et al. 2017; Cooke et al. 2020; Jain et al. 2021; Ong et al. 2022). In addition, differences in physiological tolerance to temperature and light of different Symbiodiniaceae species also results in localised adaptation and variation in the community composition of Symbiodiniaceae among populations at different latitudes (Huang et al. 2011; Silverstein et al. 2011; Chen et al. 2019; Terraneo et al. 2019). In sympatric populations where there is an absence of geographical boundaries, marine species often diverge across environmental boundaries (Forsman et al. 2020). Environmental factors that can influence coral‐Symbiodiniaceae associations in sympatry include turbidity (LaJeunesse et al. 2010; Picciani et al. 2016; Chen et al. 2019), depth (Winters et al. 2003; Iglesias‐Prieto et al. 2004; Mass et al. 2007; Sampayo et al. 2007; Byler et al. 2013) and sun exposure (Ulstrup and Van Oppen 2003; Tan et al. 2020).

An additional factor that could influence coral‐Symbiodiniaceae associations that has received little attention is differences in the timing of reproduction of the coral host. On many Western Australian reefs, there are two mass‐spawning events each year (autumn and spring) with most broadcast‐spawning corals participating in one or the other (Rosser and Gilmour 2008; Gilmour, Speed, and Babcock 2016). Genetically distinct cohorts of colonies spawning either in spring or autumn exist in multiple *Acropora* species (Rosser 2015, 2016; Gilmour et al. 2016; Rosser et al. 2017), resulting from each individual colony spawning at the same time each year. As photosynthetically derived carbon from Symbiodiniaceae contributes substantially to energy reserves required for gametogenesis (Padilla‐Gamiño et al. 2013; Rodrigues and Padilla‐Gamiño 2022; Jaffe et al. 2023), variation between Symbiodiniaceae species/genotypes in light harvesting efficiency and ability to transfer energy to the coral host (Robison and Warner 2006; Ragni et al. 2010; Karim et al. 2015; Suggett et al. 2015; terHorst and Coffroth 2022) could affect the energy available to coral hosts for reproduction. Thus, Symbiodiniaceae strains more efficient in light acquisition and carbon transfer may allow their hosts to reproduce during times where environmental conditions otherwise limit their energy resources.

To understand how Symbiodiniaceae diversity impacts coral host traits, we must be able to accurately assess Symbiodiniaceae diversity. The multi‐copy internal transcribed spacer 2 (ITS2) region of rDNA is the most commonly used marker for resolving Symbiodiniaceae species or types (Stat et al. 2012). Due to the multi‐copy nature of this region, individual Symbiodiniaceae cells often contain more than one ITS2 sequence type. This, along with the intragenomic variability of the ITS2 region, can lead to difficulty in interpreting diversity among coral samples. Different combinations of ITS2 sequences are known as ITS2 profiles, which are used to distinguish between types or species. However, some Symbiodiniaceae species share ITS2 sequences, and this is problematic when assessing the diversity of species in Symbiodiniaceae communities within a coral colony (Thornhill, LaJeunesse, and Santos 2007). This makes it difficult to interpret Symbiodiniaceae composition without knowing how many individual species or types are present in an assemblage. Increasing sampling effort has been shown to increase the number of reported ITS2 sequences, which further complicates the marker's use (Stat et al. 2011). Next‐generation sequencing methods such as 454 pyrosequencing have been used to detect Symbiodiniaceae operational taxonomic units at a significantly higher sensitivity to traditional ITS2 methods (Quigley et al. 2014; Thomas et al. 2014). More modern whole genome sequencing technologies such as Illumina provide higher throughput and greater accuracy with genome‐wide insights, which can potentially reveal even finer‐scale differences between groups (Zhang 2023).

The aim of this study was to investigate genetic differences between Symbiodiniaceae populations using whole genome sequencing to determine whether this method could detect fine‐scale genetic differences among coral host populations in Western Australia and provide new insight into factors influencing coral‐Symbiodiniaceae associations. Currently, other methods have not detected significant genetic differences in Symbiodiniaceae populations within *Acropora* species in Western Australia. For example, Thomas et al. (2014) found no difference in symbiont populations in *Acropora* assemblages between geographic regions in Western Australia using 454 amplicon sequencing. Similarly, Gilmour, Speed, and Babcock (2016); Gilmour et al. (2016) found no difference in Symbiodiniaceae populations between spring‐ and autumn‐spawning colonies of *Acropora tenuis* in Western Australia using ITS2 profiling. The objectives of this study were to investigate genetic structure of Symbiodiniaceae populations using whole genome sequencing in (1) sympatric populations of *Acropora cf. secale* that reproduce in different seasons (autumn and spring) at Ningaloo Reef and (2) geographically distinct (allopatric) populations of *Acropora millepora* separated by 11° of latitude that also spawn in different seasons (autumn and spring).

## 2 Materials and Methods

### 2.1 Study Sites

*Acropora millepora* samples were collected from a single site (~25 m radius) at each location—northern populations (*n* = 16) at Ashmore Reef (12.2686° S, 123.0539° E) in Western Australia (WA), and southern populations (*n* = 16) at Ningaloo Reef (23.1419° S, 113.7564° E) in WA. *Acropora cf. secale* samples were all collected from a single site (~25 m radius) at Ningaloo Reef, but from two different reproductive cohorts, a cohort that spawned in spring (*n* = 5) and a cohort that spawned in autumn (*n* = 5). Reproductive sampling was conducted in both species in autumn and spring, whereby colonies were examined in situ for the presence of mature eggs prior to the upcoming spawning event and identified as an autumn or spring spawner (after Baird, Guest, and Willis 2009). Each sample was stored in 95% ethanol, which was replaced after 24 h and again after 1 week. The samples were stored at room temperature before DNA extraction was performed. DNA was extracted from branch tips (without prior enrichment for Symbiodiniaceae) using DNeasy DNA extraction kits for animal tissue (Qiagen, USA) following the manufacturer's instructions.

### 2.2 Genome Sequencing

A low‐coverage whole genome sequencing approach was used in this study. For all *A. millepora* samples, paired‐end 150 bp Illumina sequencing was performed across a single lane on a NovaSeq 6000 at the Ramaciotti Centre (UNSW, Sydney). For all *A. cf. secale* samples, paired‐end 150 bp MGI sequencing was performed across three lanes at BGI Hong Kong on a DNBseq PE150 platform. To prepare the samples for further analysis, the data were demultiplexed into FASTQ files using the tool bcl2fastq2. Each sample contained reads from both the coral host and any Symbiodiniaceae present at the time of sampling (along with minor contributions from other microbial life living in the coral).

All analysis (unless otherwise specified) was done remotely using the High‐Performance Computer at the Texas Advanced Computing Centre (TACC) in the United States. To determine whether there were any issues with the quality of the sequences, the tool FastQC (Andrews 2010) was used. Each sample was analysed using this tool, and then MultiQC (Ewels et al. 2016) was used to consolidate the results from the FastQC analysis.

To increase the quality of the reads and remove any remaining adapters, low‐quality (less than Q = 15) forward and reverse reads were removed using *cutadapt* (Martin 2011). *Cutadapt* was also used to remove reads that were too short (< 25 base pairs). The trimmed samples were subsequently aligned to a custom genome containing both *Acropora millepora* (Fuller et al. 2020) and four Symbiodiniaceae genomes (one each from genera *Symbiodinium* (Shoguchi et al. 2018), *Breviolum* (Shoguchi et al. 2015), *Cladocopium* (Shoguchi et al. 2018) and *Durusdinium* (Shoguchi et al. 2020) using *bowtie2* (Langmead and Salzberg 2012)). SAMtools was then used to create new files containing only the reads which had aligned to the Symbiodiniaceae genomes, to be used in any analysis concerning variation in the Symbiodiniaceae. SAMtools was subsequently used to create files containing only the reads which had aligned to the *A. millepora* genome, to be used in any analysis concerning variation in host genes.

### 2.3 Symbiodiniaceae Identification

A custom perl script zooxType.pl. (Manzello et al. 2019) was used to count the relative proportion of reads mapping to each Symbiodiniaceae genome (from genera *Symbiodinium*, *Breviolum*, *Cladocopium* and *Durusdinium*). All samples contained predominantly Symbiodiniaceae from genus *Cladocopium*, and therefore all downstream Symbiodiniaceae analysis was focused on within‐genus diversity, using only reads which had mapped to the *Cladocopium* genome.

### 2.4 Population Structure of Host and Symbiodiniaceae

To estimate genotype likelihoods, ANGSD (Korneliussen, Albrechtsen, and Nielsen 2014) was used, as it is the most widely accepted programme for analyses involving low‐coverage population genomic data (Therkildsen and Palumbi 2017; Matz 2018). ANGSD utilises ‘soft calling’ to estimate genotype likelihoods by incorporating statistical uncertainties due to potential sequencing errors and missing data in genomic datasets with low coverage (Kim et al. 2011). ANGSD was run with the following filters: minimum mapping quality scores of 20, minimum base quality scores of 20, *p*‐value that an SNP is true of 10^−5^, minimum *p*‐value for strand bias of 10^−5^, at least 80% of non‐missing genotypes across samples, and a filter that removed any tri‐allelic SNPs.

ANGSD was used to produce two identity‐by‐state (IBS) matrices—one using only host reads and one using only Symbiodiniaceae reads. All further analysis was performed on both matrices, to identify population structure of host colonies, and their Symbiodiniaceae communities. The IBS approach compares individuals by calculating the proportion of times that reads at a certain locus are the same or different between the individuals. This method is robust to variation in sequencing coverage among samples (Korneliussen, Albrechtsen, and Nielsen 2014). The pairwise IBS matrices were provided to the function *hclust* in RStudio (version 2022.02.2 + 485) to calculate a cluster dendrogram to identify any naturally occurring clones showing genetic similarity as high as technical clones produced for this purpose. None of the samples appeared to be clones, so the same IBS matrix was used for further analysis.

To visualise any population structure present, a principal coordinates analysis (PCoA) was performed using the function *capscale* from the *vegan* package in R, using the IBS matrix as an input. To statistically assess the genetic variation between sample groups, permutational multivariate analysis of variance (PERMANOVA) based on distance matrices was used. This analysis was done using RStudio (version 2022.02.2 + 485) using the function *adonis2* from R package *vegan*, with the IBS matrix as the input.

To calculate *F*_st_ values between populations in each species, ANGSD was used to calculate .saf files for each population. Consequently, *realSFS* (a programme within ANGSD) was used to calculate site frequency spectra for each pair, and to extract the *F*_st_ values between each population pair of interest.

## 3 Results

We used whole genome sequencing to quantify genomic variation and Symbiodiniaceae associations in a final 34 samples (after poor quality sequences were removed), comprising 24 samples of *A. millepora* (14 autumn spawners from Ningaloo Reef and 10 spring spawners from Ashmore Reef) and 10 samples of *A. cf. secale* (five autumn and five spring spawners). Based on parameters such as read length, total number of reads and genome size, the average coverage of *A. millepora* samples was 1.8×, and the average coverage of *A. cf. secale* samples was 2.1×. The FastQC analysis did not flag any samples as poor quality, with every position along all reads being above the threshold of *Q* = 30. Over 99% of reads remained after quality trimming with *cutadapt* (using a quality score > 15), with mapping resulting in an alignment rate of > 95% across all samples.

### 3.1 Population Structure of Host

**FIGURE 1:**
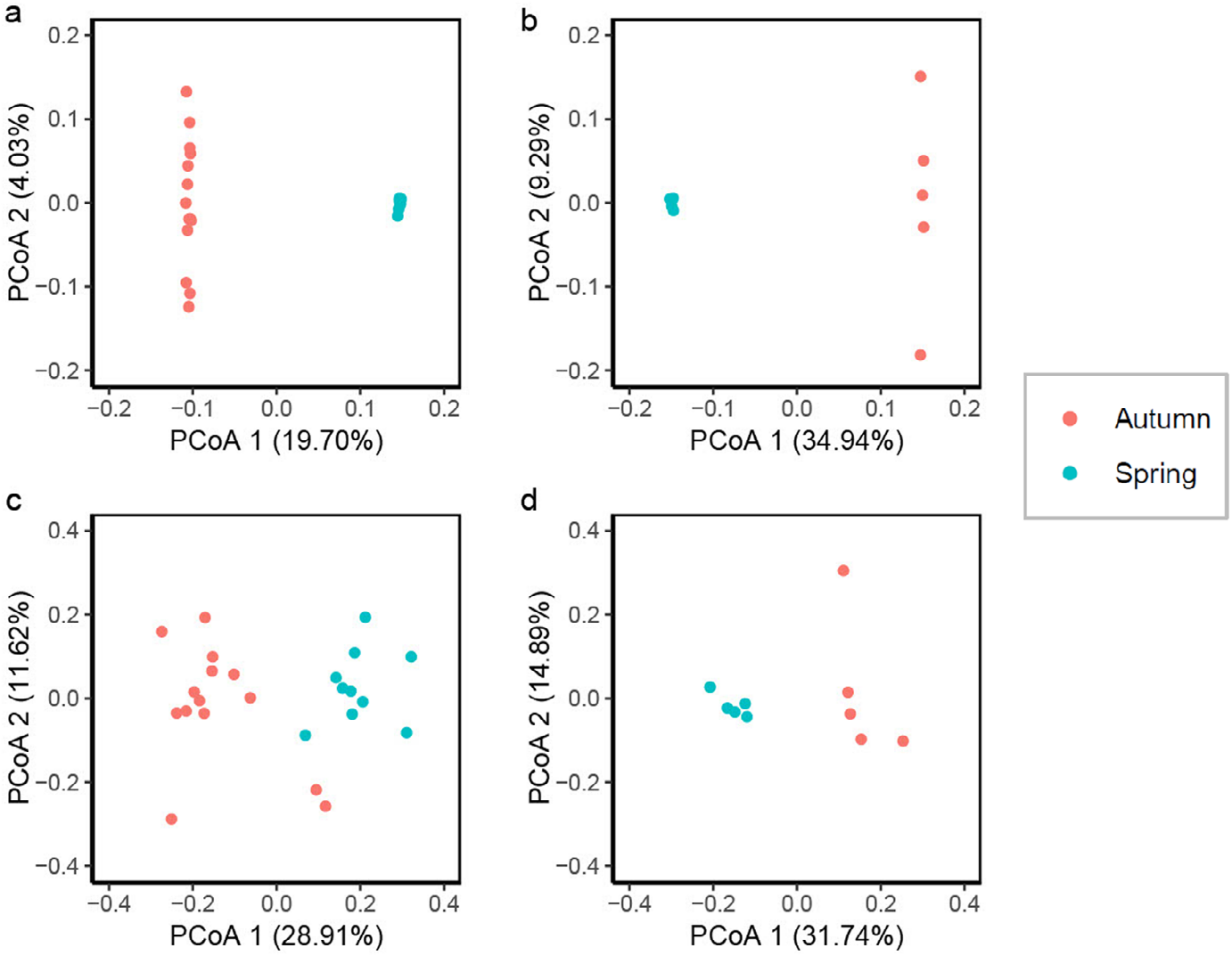
Results of principal coordinates analysis (PCoA) showing the first two principal coordinates, and the percentage of variation explained by each axis. PCoA based on IBS matrix of genomic distance between (a, b) coral hosts in (a) allopatric populations of *Acropora millepora*, (b) sympatric populations of *A. cf. secale*, and (c, d) *Cladocopium* Symbiodiniaceae in (c) allopatric populations of *A. millepora* , (d) sympatric populations of *A. cf. secale*.

A PCoA of genome‐wide variation showed clear genetic differentiation between spring‐spawning *A. millepora* colonies from Ashmore Reef and autumn‐spawning *A. millepora* colonies from Ningaloo Reef (Figure 1a). This result was supported by a PERMANOVA which showed that geographic location (or alternatively, spawning time) had a significant effect on genetic variation between these groups (Adonis, *R* ^2^ = 0.1970, *p* = 0.001). A PCoA also showed genetic differentiation between sympatric spring and autumn spawners of *A. cf. secale* (Figure 1b). Similarly, this was supported by PERMANOVA results showing significant genetic variation between spring‐ and autumn‐spawning groups in *A. cf. secale* (Adonis, *R* ^2^ = 0.3494, *p* = 0.01). This high level of genetic variation between populations within species was also supported by genome‐wide estimates of *F*_st_ ( *A. millepora* *F*_st_ = 0.112; *A. cf. secale F*_st_ = 0.112).

### 3.2 Population Structure of Symbiodiniaceae

**FIGURE 2:**
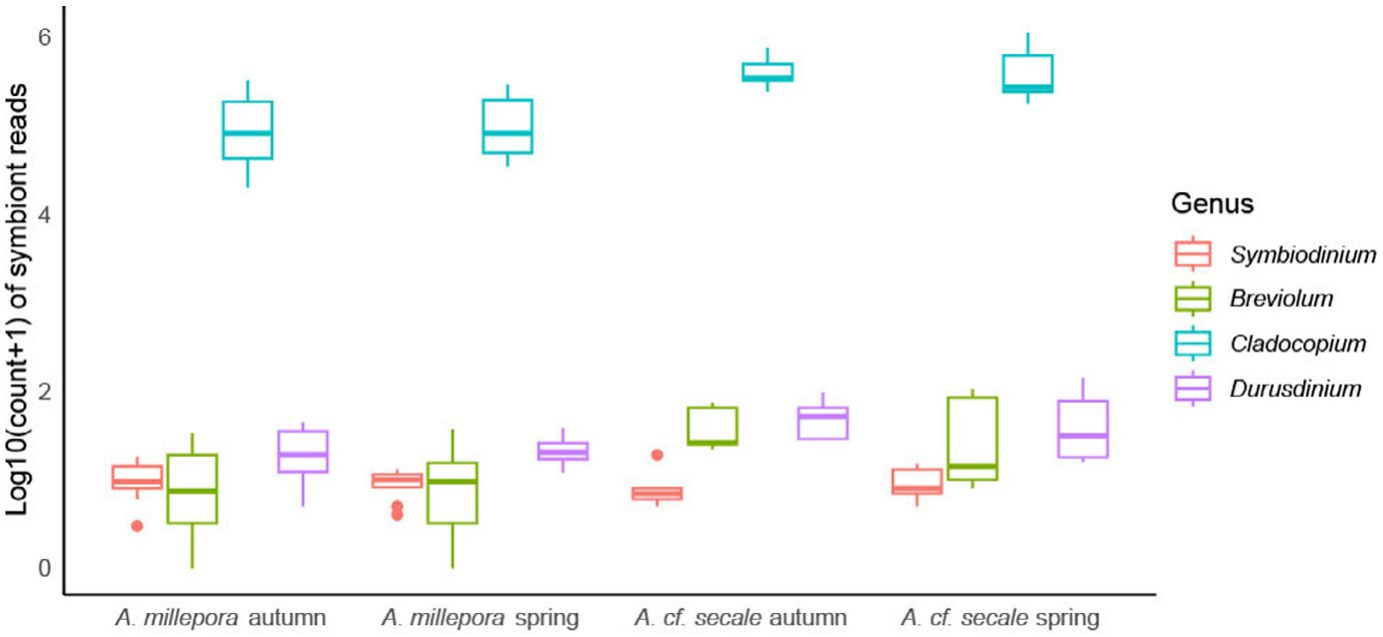
Box plot showing log_10_ of number of reads assigned to each genus of Symbiodiniaceae in four sample groups (*Acropora cf. secale* autumn spawners, *A. cf. secale* spring spawners, *Acropora millepora* autumn spawners, *A. millepora* spring spawners). Line inside box represents median, and dots represent outliners.

Based on the relative proportion of reads classified as Symbiodiniaceae, all samples from both species were found to contain predominantly Symbiodiniaceae from the genus *Cladocopium*, with very minor contributions from other genera (Figure 2). A PCoA of genome‐wide variation showed clear genetic differentiation of Symbiodiniaceae populations between spring‐spawning *A. millepora* colonies from Ashmore Reef and autumn‐spawning *A. millepora* colonies from Ningaloo Reef (Figure 1c). This result was supported by a PERMANOVA which showed that geographic location (alternatively spawning time) had a significant effect on genetic variation of Symbiodiniaceae populations between these groups (Adonis, *R* ^2^ = 0.2616, *p* = 0.001). A PCoA also showed genetic differentiation between sympatric spring and autumn spawners of *A. cf. secale* (Figure 1d). Similarly, this was supported by PERMANOVA results showing significant genetic variation of Symbiodiniaceae among spring‐ and autumn‐spawning groups in *A. cf. secale* (Adonis, *R* ^2^ = 0.3029, *p* = 0.015).

## 4 Discussion

This study found significant genetic differences in Symbiodiniaceae populations between geographic regions and spawning seasons, demonstrating that this novel analysis using whole genome sequencing improved the sensitivity to detect genetic population structure within species that has not been detected in other studies (e.g. Thomas et al. 2014; Gilmour, Speed, and Babcock 2016; Gilmour et al. 2016). We show that this method can reveal fine‐scale patterns of differentiation among Symbiodiniaceae populations, based on relatively small genetic differences, improving our ability to identify genetic and potentially functional differences associated with variation in Symbiodiniaceae populations, and furthering our understanding of this critical symbiotic relationship.

In this study, we found a significant genetic differences in Symbiodiniaceae hosted by *Acropora millepora* cohorts separated by 11° of latitude that spawn in different seasons (Ashmore Reef colonies spawn in spring, and Ningaloo Reef colonies spawn in autumn). The effects of location and spawning season could not be disentangled in our analysis, so we are unable to say whether one factor had a greater effect than the other. Significant differences in Symbiodiniaceae communities between geographical populations have been observed in other studies (van Oppen et al. 2001; Ziegler et al. 2017; Cooke et al. 2020; Jain et al. 2021; Ong et al. 2022) and have been attributed to limited dispersal (Santos, Gutierrez‐Rodriguez, and Coffroth 2003; Thornhill et al. 2009; LaJeunesse et al. 2010), the light environment (Finney et al. 2010; Eckert et al. 2020; Kaniewska and Sampayo 2022), and differences in thermal tolerance (Howells et al. 2012; Byler et al. 2013; Pettay and LaJeunesse 2013). These factors could be contributing to the genetic differences between Symbiodiniaceae populations in *A. millepora* populations here. Interestingly, however, there was also a significant genetic difference between Symbiodiniaceae populations in sympatric populations of *Acropora cf. secale* that reproduce in different seasons (autumn and spring), suggesting that differences in spawning time could also play a role in *A. millepora* .

Sympatric populations of *A. cf. secale* are theoretically exposed to a common pool of Symbiodiniaceae as adults. However, the results of this study show that these corals associate with distinct genotypes, strongly suggesting that there is host‐Symbiodiniaceae specificity associated with differences in the timing of reproduction by the coral host. Though the populations of *A. cf. secale* studied here come from the same environment, the temporal separation in their time of reproduction each year (spring or autumn) means that each is exposed to different environmental conditions as they undergo gametogenesis. Gametogenesis requires a large energy investment from coral colonies (Gomez et al. 2018), particularly in the form of lipids which are incorporated into gametes to provide an energy resource for offspring (Padilla‐Gamiño et al. 2013) and photosynthetically derived carbon from Symbiodiniaceae that contribute greatly to energy reserves in gamete bundles (Rodrigues and Padilla‐Gamiño 2022; Jaffe et al. 2023). Thus, differences in photosynthetic efficiency (terHorst and Coffroth 2022) and ability to transport autotrophic carbon to the coral host (Pernice et al. 2015) between Symbiodiniaceae species or genotypes could lead to variation in the energetic budget available for coral reproduction. This could lead to host‐Symbiodiniaceae specificity, with natural selection encouraging association with Symbiodiniaceae genotypes that contribute more energy to the holobiont during these times of high energy demand, particularly to spring spawners which require more energy to sustain gametogenesis over winter. In addition, differentiation of autumn and spring Symbiodiniaceae populations may also be driven by genetic differentiation of their host populations, as has been observed in other studies (Santos et al. 2004; Prada et al. 2014). Importantly, this can be due to genetic drift influencing host‐Symbiodiniaceae recognition, without natural selection for metabolic adaptations. The importance of this neutral process relative to the adaptive process generating different metabolic phenotypes would be a great subject for future research.

## Author Contributions

**Sanna Y. Eriksson:** conceptualization (equal), formal analysis (lead), investigation (lead), project administration (lead), writing – original draft (lead), writing – review and editing (equal). **Mikhail V. Matz:** methodology (supporting), resources (lead), software (lead), supervision (supporting), writing – review and editing (supporting). **Peter D. Vize:** methodology (supporting), supervision (supporting), writing – review and editing (supporting). **Natalie L. Rosser:** conceptualization (equal), data curation (lead), funding acquisition (lead), supervision (lead), writing – review and editing (equal).

## Conflicts of Interest

The authors declare no conflicts of interest.

## Data Availability

Scripts and code available at: https://github.com/sanna2110/SYMBIO_WA. Raw reads available at BioProject: PRJNA1098905. Other data: https://datadryad.org/stash/share/Y58vw5M5VOg45qhYINXHE6dieGMTX3LFcUO1U3jcDio.
